# Incident Hip Fractures among Community Dwelling Persons with Alzheimer’s Disease in a Finnish Nationwide Register-Based Cohort

**DOI:** 10.1371/journal.pone.0059124

**Published:** 2013-03-18

**Authors:** Anna-Maija Tolppanen, Piia Lavikainen, Hilkka Soininen, Sirpa Hartikainen

**Affiliations:** 1 Institute of Clinical Medicine – Neurology, University of Eastern Finland, Kuopio, Finland; 2 Kuopio Research Centre of Geriatric Care, University of Eastern Finland, Kuopio, Finland; 3 Department of Neurology, Kuopio University Hospital, Kuopio, Finland; 4 Pharmacology and Geriatric Pharmacotherapy Unit, University of Eastern Finland, Kuopio, Finland; UCSD School of Medicine, United States of America

## Abstract

**Background:**

Previous cohort studies have shown that persons with Alzheimer’s disease (AD) have a higher risk of hip fractures but recent data from large representative cohorts is scarce.

**Methods:**

We investigated the association between AD and prevalent and incident hip fractures in an exposure-matched cohort study conducted in Finland 2002–2009 (the Medication and Alzheimer’s disease in 2005 study; MEDALZ-2005). The study population included all community-dwelling persons with verified AD diagnosis in Finland on December 31, 2005 and one matched comparison person per AD case (N = 56,186, mean age 79.9 (SD 6.8) years, range 42–101 years). The diagnosis of AD was extracted from a special reimbursement register. Data on hip fractures during 2002–2009 was extracted from the Finnish National hospital discharge register. Analyses of incident hip fractures (n = 2,861) were restricted to years 2006–2009.

**Results:**

Persons with AD were twice as likely to have previous hip fracture in 2005 (odds ratio, 95% confidence interval 2.00, 1.82–2.20) than matched aged population without AD. They were also more likely to experience incident hip fracture during the four-year follow-up (hazard ratio, 95% confidence interval 2.57, 2.32–2.84, adjusted for health status, psychotropic drug and bisphosphonate use). The AD-associated risk increase decreased linearly across age groups. Although people with AD had higher risk of hip fractures regardless of sex, the risk increase was larger in men than women.

**Conclusion:**

Findings from our nationwide study are in line with previous studies showing that persons with AD, regardless of sex or age, have higher risk of hip fracture in comparison to general population. Although there was some suggestion of effect modification by age or sex, AD was consistently associated with doubling of the risk of incident hip fracture.

## Introduction

Alzheimer’s disease (AD) is the most common form of dementia. Although the neuropsychiatric symptoms themselves affect the quality of life, individuals with AD are at increased risk for other comorbidities which further decrease the individuals’ well-being and increase the healthcare costs. Persons with cognitive impairment [1], [2] and dementia, including AD [3] have higher risk of falls. Individual cohort studies, [1], [4]–[12] analyses based on US Medicare claims data [13], [14], UK electronic database of primary care data [15] and a recent meta-analysis [16] have shown that individuals with AD have 2–3 fold risk of hip fracture in comparison to dementia-free individuals. Importantly, persons with AD also have worse prognosis after hip fracture [17]: they are more likely to be immobilized [18] and have higher post-fracture mortality [15].

Majority of the previous studies have been conducted in restricted cohorts [1], [4]–[12] and one of the three larger studies [13]–[15] is based on data from the mid-1990s [13]. Thus, recent data from a large representative cohort is scarce. Higher incidence of hip fractures, especially in combination with worse outcomes following the hip fractures would emphasize the need for effective hip fracture prevention strategies. We investigated the prevalence and incidence of hip fractures among people with AD in a register-based nationwide cohort including all non-institutionalized persons with AD residing in Finland in 2005.

## Participants and Methods

### Study Cohort

**Figure 1:**
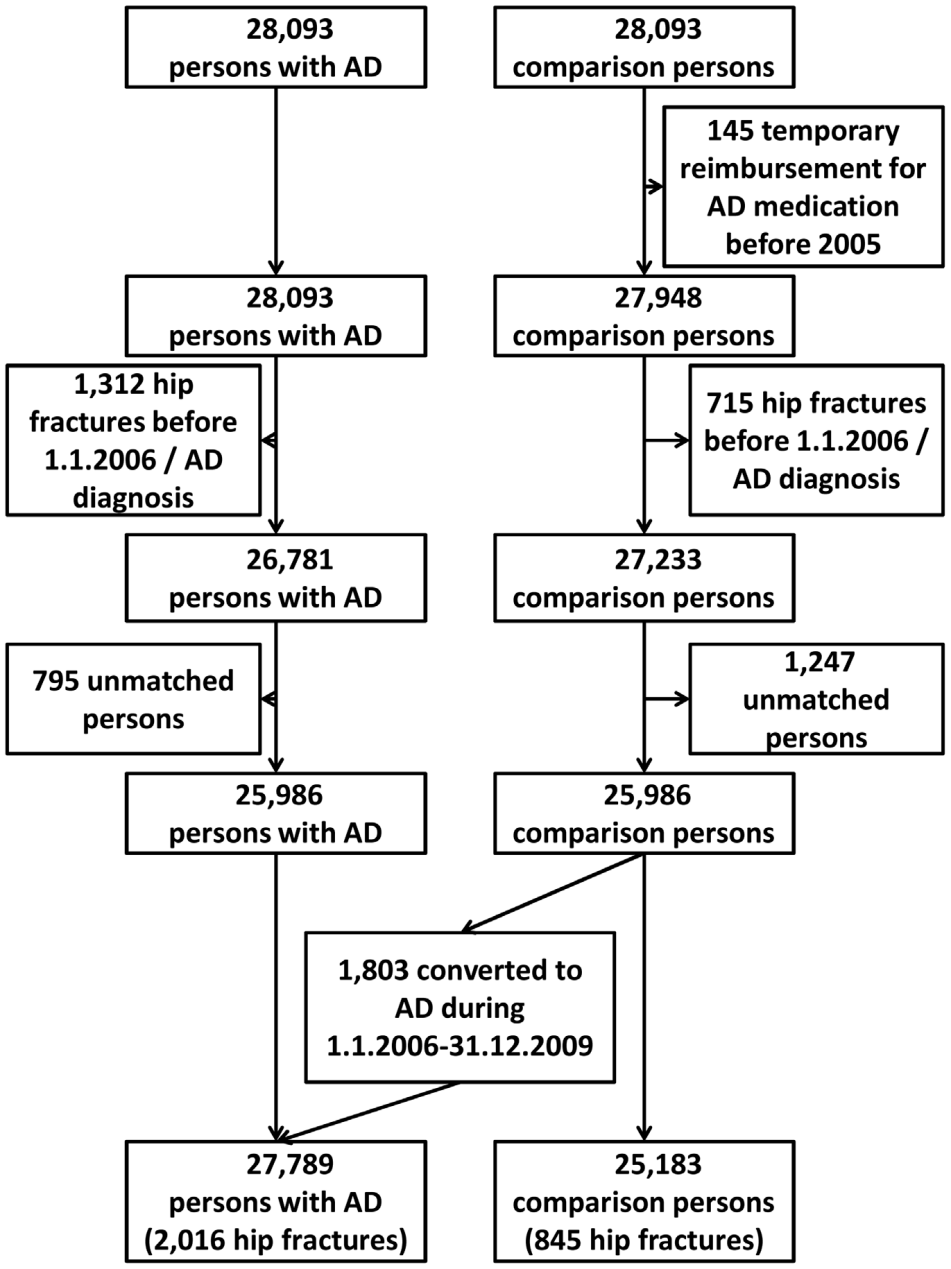
Formation of study cohort.

Retrospective (years 2002–2005) and prospective (years 2006–2009) data for the Medication and Alzheimer’s Disease (MEDALZ-2005) study were used. The MEDALZ-2005 cohort includes all community-dwelling persons with a verified diagnosis of Alzheimer’s disease residing in Finland on 31 December 2005 (n = 28,093) and a single age-, sex- and region of residence- matched comparison person for each individual with AD (N = 56,186). Altogether 2,015 comparison persons converted to AD during the four-year follow-up on 2006–2009. The age range of the cohort was 42–101 years (mean 79.9 (SD 6.8) years) and 38,086 (67.8%) of the sample were women. AD diagnosis was based on the NINCS-ADRDA and DSM-IV criteria for Alzheimer’s disease.{[19], [20] Persons with AD were identified from the Special Reimbursement Register maintained by the Social Insurance Institution of Finland (SII). The Special Reimbursement Register contains records of persons who are eligible to receive reimbursement due to e.g. AD, cancer or diabetes. The comparison persons were identified from the register that contains all residents of Finland who are entitled to benefits by the Social Insurance Institution (SII). Each resident of Finland is assigned a unique social security number which was used to track participant data and link data from different national registers of hospital discharges, prescriptions and mortality. Linking was performed by SII and all data were de-identified before submission to the research team. No ethics committee approval was required as only de-identified data were used and the study participants were not contacted. Formation of the study sample is described in Figure 1.

### Outcomes

Hip fractures during 2002–2009 were identified from the national hospital discharge register based on the following ICD-10 codes: S72.0 (fracture of neck of femur), S72.1 (**pertrochanteric fracture**) and S72.2 (s**ubtrochanteric fracture**). Finnish hospital discharge register is a statutory register containing information on use of in- and outpatient healthcare services. The individual-level data is collected and updated continuously.

### Confounders

Information on cardiovascular diseases, cancer, diabetes (type 1 or 2), pernicious anemia and other disturbances in absorption of vitamin B_12_, Parkinson’s disease, epilepsy, glaucoma and rheumatoid conditions was obtained from Finnish National Prescription Register. Details are given in Supplementary Table 1 (see file Table S1). To account for medications which may affect the susceptibility to hip fractures, we adjusted for use of psychotropic drugs (The Anatomical Therapeutic Chemical: ATC Classification system classes N05, N06A and N06B) and bisphosphonates (ATC classes M05BA and M05BB). Psychotropic and bisphosphonate use were defined as at least one filled prescription during 2005. Data on filled prescriptions were obtained from the National Prescription register, which contains records of prescription drugs purchased by all Finnish residents living in non-institutionalized settings.

**Table 1:** Association of Alzheimer’s disease with hip fractures.

Agegroup	AD			No AD			HR (95%CI)		
	N	n of events (%)	events/1,000 person-years	N	n of events (%)	events/1,000 person-years	Model 1^a^	Model 2^b^	Model 3^c^
<80	12,851	744 (5.8)	18	11,611	199 (1.7)	4	4.27 (3.59, 5.08)	4.51 (3.72, 5.48)	4.10 (3.36, 5.00)
80–84.9	8,794	693 (7.9)	27	7,290	328 (4.5)	12	2.34 (2.01, 2.72)	2.43 (2.07, 2.86)	2.37 (2.00, 2.81)
≥85	6,144	579 (9.4)	37	5,282	318 (6.0)	19	1.98 (1.68, 2.32)	1.93 (1.63, 2.29)	1.80 (1.51, 2.15)
All	27,789	2,016 (7.3)	24	24,183	845 (3.5)	10	2.70 (2.47, 2.96)	2.74 (2.48, 3.03)	2.57 (2.32, 2.84)

a Unadjusted.

b Adjusted for cardiovascular diseases, cancer, diabetes, pernicious anemia and other disturbances in absorption of vitamin B_12_, Parkinson’s disease, epilepsy, glaucoma and rheumatoid arthritis.

c Adjusted for covariables in Model 2 plus use of bisphosphonate and psychotropic drugs in 2005.

### Statistical Analyses

All statistical analyses were conducted with Stata 12.0 (Stata Corp LP, College Station, TX USA). Association between prevalent hip fractures before AD diagnosis was assessed with conditional logistic regression. Figure 1 shows how the study population for incident hip fractures after AD diagnosis was derived. Due to the cohort definition, January 1^st^ 2006 was set as a beginning of follow-up and participants who had experienced hip fracture before beginning of follow up or AD diagnosis were excluded from the incidence analyses (n = 2,027). In addition 145 comparison persons had temporarily been entitled to reimbursed AD medication before 2005 and were excluded. Because our study is an exposure-matched cohort (with AD as an exposure), we excluded those persons whose referent was excluded, leaving 25,986 AD-comparison person pairs. Altogether 1,803 comparison persons converted to AD during the follow-up and thus AD was modeled as a time-dependent covariate.

Proportional hazards for incident hip fractures were estimated with Cox regression. Matching was taken into account in the analysis. We present hazard rations for the whole study population and due to interaction (P for interaction with sex = 0.05 and P for interaction with age<0.001), sex- and age group-wise hazard ratios are also presented. Chronological age was categorized as <80, 80–84 and ≥85 years. We also present the results according to quartiles of approximate age at Alzheimer’s disease diagnosis, calculated from the diagnosis of AD or first reimbursed purchase of AD medication, depending on which one occurred first. The cutoffs for classes of age at AD diagnosis were 74, 79 and 83 years, resulting to the following categories: 38.9–73.9, 74–78.9, 79.0–82.9 and 83.0–99.0 years.

Differences in the mean age of first fracture between persons with and without AD were estimated with t-test. The age at first fracture was normally distributed.

## Results

The prevalence of previous hip fractures in the full study cohort (N = 56,186) in December 2005 was 4.7% (n = 1,312) in persons with AD and 2.4% (n = 667) in those without AD. These individuals, together with 48 comparison persons who experienced a hip fracture before they converted to AD during the follow-up were excluded from the analysis of incident hip fractures. Persons with AD were more likely to have previous hip fractures (OR, 95% CI 2.00, 1.82–2.20) and they had experienced the fractures at younger age than those without Alzheimer’s disease (difference for the age at hip fracture 1.25 years, 95% CI 0.72–1.79 years). The mean age at fracture was 81.6 (SD 5.8) years for cases and 82.8 (SD 5.6) years for controls (P for difference <0.0001).

Altogether 2,861 persons (2,016 with AD and 845 with no AD) experienced an incident hip fracture during 2006–2009. The follow-up time was 87,888 person-years in the AD group and 83,324 years in the non-AD group.

Table 1 shows the association between AD and incident hip fractures. In the crude analyses, persons with Alzheimer’s disease were 2.7 times more likely to experience incident hip fractures during 2006–2009 than persons without AD (Model 1). Adjustment for comorbidities resulted in a modest strengthening in the association in the whole population and two youngest age groups while an opposite trend was observed in the oldest age group (Model 2). Further adjustment for the use of psychotropic drugs or bisphosphonates attenuated the association in all age groups (Model 3) but in the whole study population, the relative risk of hip fracture in the AD group was still 2.6 times higher in comparison to persons without AD. The magnitude of the association was stronger in the youngest age group, although AD was consistently associated with higher risk of incident hip fractures in all age groups.

Table 2 shows the association between AD and incident hip fractures in men and women. Although people with AD had higher risk of hip fractures regardless of sex, the risk increase was slightly larger in men than women when all age groups were pooled in the analyses. In the youngest age group (<80 years, the risk increase was larger in women than men while an opposite pattern was observed in the two oldest age groups (age 80–84 years and age ≥86 years).

**Table 2:** Association of Alzheimer’s disease with hip fractures in men and women.

Age group	AD cases			Non-AD			HR (95%CI)		
	N	n of events (%)	events/1,000 person-years	N	n of events (%)	events/1,000 person-years	Model 1^a^	Model 2^b^	Model 3^c^
Men									
<80	4,956	195 (3.9)	13	4,526	66 (1.5)	4	3.71 (3.22, 4.27)	3.85 (3.30, 4.50)	3.51 (2.99, 4.12)
80–84.9	2,655	167 (6.3)	23	2,243	62 (2.8)	8	2.51 (2.21, 2.85)	2.53 (2.21, 2.90)	2.41 (2.09, 2.77)
≥85	1,565	117 (7.5)	31	1,385	53 (3.8)	13	2.33 (2.04, 2.65)	2.31 (2.01, 2.65)	2.14 (1.86, 2.47)
All	9,176	479 (5.2)	18	8,154	181 (2.2)	6	2.92 (2.41, 3.54)	2.94 (2.38, 3.63)	2.70 (2.18, 3.36)
Women									
<80	7,895	549 (7.0)	21	7,085	133 (1.89)	5	2.71 (2.45, 2.99)	2.75 (2.47, 3.05)	2.57 (2.30, 2.87)
80–84.9	6,139	526 (8.6)	28	5,047	266 (5.3)	14	2.66 (2.41, 2.94)	2.74 (2.46, 3.05)	2.58 (2.31, 2.89)
≥85	4,579	462 (10.1)	38	3,897	265 (6.8)	21	2.63 (2.37, 2.90)	2.67 (2.40, 2.98)	2.52 (2.25, 2.82)
All	18,613	1,537 (8.3)	27	16,029	664 (4.1)	11	2.64 (2.38, 2.93)	2.71 (2.42, 3.03)	2.55 (2.27, 2.87)

a Unadjusted.

b Adjusted for cardiovascular diseases, cancer, diabetes, pernicious anemia and other disturbances in absorption of vitamin B_12_, Parkinson’s disease, epilepsy, glaucoma, rheumatoid arthritis.

c Adjusted for covariables in Model 2 plus use of bisphosphonate and psychotropic drugs in 2005.

Association between AD and hip fracture according to AD duration are shown in Table 3. In comparison to those with no AD, risk of hip fractures was highest among those who were youngest when AD was diagnosed (38.9–73.9 years) and the relative risk difference decreased across categories of age at AD diagnosis. These findings were in line with the pattern observed with chronological age (Table 1).

**Table 3:** Hazard ratios for hip fractures according to age at AD diagnosis.

Age at diagnosis	N	n of events (%)	events/1,000 person-years	HR (95%CI)		
				Model 1^a^	Model 2^b^	Model 3^c^
No AD	24,183	845 (3.5)	10	1.00 (reference)	1.00 (reference)	1.00 (reference)
<74 years	6,871	347 (5.1)	15	5.60 (4.28, 7.33)	5.64 (4.29, 7.41)	5.11 (3.87, 6.74)
74.0–78.9 years	7,621	542 (7.1)	22	3.19 (2.67, 3.81)	3.20 (2.66, 3.84)	2.97 (2.46, 3.57)
79.0–82.9 years	6,546	526 (8.0)	26	2.14 (1.82, 2.51)	2.17 (1.84, 2.56)	2.04 (1.73, 2.41)
≥83 years	6,751	601 (8.9)	31	1.75 (1.50, 2.05)	1.78 (1.51, 2.08)	1.68 (1.43, 1.98)

a Unadjusted.

b Adjusted for cardiovascular diseases, cancer, diabetes, pernicious anemia and other disturbances in absorption of vitamin B_12_, Parkinson’s disease, epilepsy, glaucoma and rheumatoid arthritis.

c Adjusted for covariables in Model 2 plus use of bisphosphonate and psychotropic drugs in 2005.

## Discussion

In this nationwide study individuals with AD had two times higher prevalence of previous hip fractures and when these individuals were excluded from the analyses, the incidence of new hip fractures was 2.6-fold in those with AD in comparison to their matched controls. These estimates are in accordance with previous studies showing a 2-3-fold increase in hip fracture risk among AD patients [1], [4], [6]–[8], [10]–[15], [21].

Strengths of our study include large, representative cohort: all AD cases entitled to special reimbursement of AD medication and residing in Finland were included. Thus our study population includes only cases that were verified by medical examination including computed tomography/magnetic resonance imaging scan, exclusion of alternative diagnoses and confirmation of the AD diagnosis by a neurologist or geriatrician. The Finnish Current Care Guidelines recommend that all persons with AD are treated with anti-dementia drugs unless there is a specific contraindication. Patients with mild or moderate AD are entitled to reimbursed anti-dementia medication, but the reimbursement is not withdrawn if/when the patient develops severe AD. Thus our study sample included persons with all stages of AD. The Finnish Hospital Discharge Register covers nearly all occurring hip fractures. A study comparing audit and register-based data showed that 98.1% of occurred hip fractures were recorded in the hospital discharge register [22]. Register-based diagnoses of dementia/AD likely underestimate the disease incidence and thus we cannot exclude the possibility that some of the persons who did not have special reimbursement for AD medication actually had AD. Thus, our results may be an underestimate of the true association although the comparison to previous studies suggests that this bias in likely to be small.

Due to the register-based approach, we were unable to adjust for lifestyle-based confounders or genetic risk factors, such as apolipoprotein E ε4-allele, which has been suggested to be a risk factor for hip fracture, independent of the effect of dementia and falling [23], [24]. However, we extracted information on co-morbidities, such as cancer, diabetes and cardiovascular diseases, which likely capture some variation in lifestyle. We acknowledge that some residual confounding is likely, although the results were in line with previous studies that were able to adjust for directly measured lifestyle factors [1], [4]–[12]. We were also able to adjust for the use of psychotropic and bisphosphonate medication on the basis of prescription data. In addition, the hospital discharge data were available from years 2002–2009 which may have affected our ability to strictly exclude all previous hip fractures. Our study was restricted to individuals who were non-institutionalized at the baseline, which may affect the generalisability of the results.

Although less than 10% of falls cause hip fracture, [25], [26] over 90% of hip fractures are due to falls [27], [28] and accordingly, a higher number of falls further increases the risk of hip fracture [29]. Accidental injuries, including falls are the most common adverse event among institutionalized AD patients [30]. Both cognitive impairment [1], [2] and different dementias, including AD [3] are associated with increased risk of falls. Consequently, in a prospective study of female patients with AD, those with falls were more likely to experience hip fractures [31]. The association between dementia and falls is not restricted to those living in institutionalized settings [3] and the increased risk of falling can be explained by gait and balance disorders, defective neuromuscular regulation, visual disorders, postural hypotension, depression, low body mass index or use of psychotropic drugs, all of which are more common among individuals with AD [32]. Surprisingly, findings from an older cohort suggest that too fast gait may increase the risk of falling: although frail patients with dementia walked slowly, they still walked relatively too fast considering the overall degree of physical impairment that should have warranted a slower gait [33]. Motor function is also affected and especially dual-tasking (i.e. performing a cognitive task while walking) is impaired already in the mild forms of AD [34].

Due to the definition of AD in our study (i.e. clinically verified diagnosis of AD), the increase in hip fractures could be due to adverse effects of drugs instead of risk mediated by the disease process. Use of acetylcholinesterase inhibitors is associated with higher rates of syncope, bradycardia, and hip fractures in older adults with dementia, [35] although a recent case-control study of 2258 AD patients (and 80 hip fractures) found an association between acetylcholinesterase inhibitor use and decreased risk of hip fracture [36]. In our study cohort, 86% of persons with AD used anti-dementia drugs and 71% of these used acetylcholinesterase inhibitors in 2005 [37]. However, the prevalence of hip fractures was higher among AD cases, suggesting that the increase could mainly be due to factors associated with the ongoing Alzheimer’s disease process, such as postural hypotension, perception difficulties or motor problems.

In conclusion, findings from this nationwide study are in line with previous studies showing that persons with AD, regardless of age, sex or disease duration have higher risk of hip fracture in comparison to the general population. As people with AD are known to have worse prognosis after hip fracture, these findings highlight the need for effective strategies to prevent hip fractures in this vulnerable population.

## Supporting Information

Table S1 **Special reimbursement codes used for extracting information on confounders.**
(DOCX)
