# Supplementary material for: Incident Hip Fractures among Community Dwelling Persons with Alzheimer’s Disease in a Finnish Nationwide Register-Based Cohort
Source: PLoS One. 2013 Mar 18;8(3):e59124. doi: 10.1371/journal.pone.0059124 (PMC3601105; doi:10.1371/journal.pone.0059124)
Supplement: Table S1 — Special reimbursement codes used for extracting information on confounders. (DOCX) [file pone.0059124.s001.docx]

**Table S1. Special reimbursement codes used for extracting information on confounders**

| Diabetes | diabetes mellitus |
| --- | --- |
| Cardiovascular diseases | chronic cardiac insufficiency  chronic hypertension  chronic coronary heart disease  chronic arrythmias |
| Pernicious anaemia | pernicious anaemia and other disturbances in vitamin B_12_ absorption |
| Parkinson’s disease | Parkinson’s disease |
| Epilepsy | epilepsy |
| Glaucoma | glaucoma |
| Rheumatic conditions | rheumatic arthritis and other rheumatic conditions |
| Cancer | breast cancer  prostate cancer  leukemia and other malignant diseases of the blood and bone marrow  cancers of female genital organs  other malignant tumours  dasatinib, nilotimib, gefitinib, pazopanib, lapatinib, imatinib, erlotinib, sunitinib and sorafenib  interferon-α treatment for melanoma for renal cancer  paclitaxel and docetaxel  fulvestrant  lenalidomid  trabectedin  everolimus |
